# The Short- and Long-Term Risk of Stroke after Herpes Zoster - A Nationwide Population-Based Cohort Study

**DOI:** 10.1371/journal.pone.0069156

**Published:** 2013-07-17

**Authors:** Nandini Sreenivasan, Saima Basit, Jan Wohlfahrt, Björn Pasternak, Tina N. Munch, Lars P. Nielsen, Mads Melbye

**Affiliations:** 1 Department of Epidemiology Research, Statens Serum Institut, Copenhagen, Denmark; 2 Department of Virology, Statens Serum Institut, Copenhagen, Denmark; Hunter College, City University of New York (CUNY), CUNY School of Public Health, United States of America

## Abstract

**Background and Objective:**

Varicella zoster virus (VZV) is known to cause VZV vasculopathy, which may be associated with stroke. A recent study found an increased risk of stroke within one year of herpes zoster. We aimed to investigate the short and long-term effects of herpes zoster on the risk of stroke.

**Methods:**

Using Danish national registers, we constructed a cohort consisting of all Danish adults ≥18 years old between 1995 and 2008 (n = 4.6 million; person-years of follow-up = 52.9 million). Individual-level information on prescriptions for herpes zoster antiviral treatment and diagnoses of stroke was obtained from national registers. We compared the risk of stroke in persons who had received the specific dosage of acyclovir for herpes zoster with persons who had never received antiviral treatment by Poisson regression.

**Results:**

During follow-up, 2.5% received treatment for herpes zoster and 5.0% were diagnosed with stroke. Individuals who had received medication had a 127% (95% CI 83–182%) increased risk the first two weeks, 17% (CI 9–24%) between two weeks and one year, and 5% (2–9%) after the first year. The increased risk was greatest in the youngest age group (<40). To control for healthcare-seeking behaviour, we conducted parallel analyses investigating the risk of selected fractures after herpes zoster and found no similar increased risks.

**Conclusions:**

This large nationwide cohort study found an increased risk of stroke after treatment for herpes zoster. Although the short-term risk was particularly high, we cannot rule out the possibility of a small but important long-term risk.

## Introduction

Approximately 15 million people worldwide suffer from a stroke every year leading to five million deaths and an additional 5 million individuals left disabled. Common risk factors for stroke include smoking, atrial fibrillation, hypertension and other cardiovascular factors. Although improved management of modifiable risk factors has reduced the burden of stroke, the total number continues to increase due to an aging population. [1].

In recent years, varicella zoster virus (VZV) has also been identified as a risk factor for stroke. Primary infection with VZV usually manifests as chickenpox during childhood. After primary infection, the virus becomes latent and can reactivate years later as herpes zoster; the risk of herpes zoster increases with age and immunosuppression.[2]–[4] Only two epidemiologic studies have investigated the incidence of stroke in adults after herpes zoster. These studies showed a 31% and 450% increased risk of stroke the first year after herpes zoster and herpes zoster ophthalmicus, respectively. [5], [6] Few studies have focused on the time lag between herpes zoster and the onset of stroke. Most reports have described the onset of neurological symptoms occurring days to months after varicella or herpes zoster, while one review of 30 cases found neurologic symptoms to occur up to 2.5 years after the rash.[7]–[11].

We conducted a nationwide population-based cohort study using the Danish national registers to investigate the risk of stroke/transient ischemic attack (TIA) after receiving systemic antiviral medication for herpes zoster, andto assess the delay between herpes zoster and development of stroke/TIA.

## Methods

### Study Cohort

We constructed a nationwide cohort consisting of all persons born in Denmark who were alive and ≥18 years between January 1, 1995 and December 31, 2008. Cohort members were identified using information from the Danish Civil Registration System (CRS). The CRS was established in 1968 and contains demographic information on all people living in Denmark. All residents of Denmark are registered in the system and are assigned a unique personal identifier (CRS-number) that allows follow-up of study subjects and linkage of information from the nationwide population-based health registers used in this study. [12].

### Ethics Statement

The study was approved by the Danish Data Protection Agency. In Denmark, ethics approval and individual consent are not required for registry-based research.

### Exposure to Antiviral Medication

In Denmark, uncomplicated herpes zoster is usually treated in the primary care sector, from which diagnostic data are not available. To identify persons with herpes zoster, we used the Danish National Register of Medicinal Product Statistics (NRMPS) to obtain information on cohort members that had received prescription drugs for herpes zoster. The NRMPS was established in 1994 and includes individual-level information on all prescriptions filled at pharmacies in Denmark, the CRS-number of the recipient, anatomical therapeutic chemical (ATC) code, drug strength, number of packages, package size, and the date on which the prescription was filled. This register is considered close to complete for prescription drugs in Denmark. [13].

In Denmark, the following systemic antiviral courses are used for the treatment of uncomplicated herpes zoster in immuno-competent persons: 1) 800 mg acyclovir, five times daily for 7 days, 2) 1 g valacyclovir, three times daily for 7 days, or 3) famciclovir, 500 mg, three times daily for 7 days. These systemic antivirals are available by prescription only. We obtained information on all acyclovir (ATC code J05AB01), valacyclovir (ATC code J05AB11) and famciclovir (ATC code J05AB09) prescriptions filled by cohort members between 1995 and 2008.

These antivirals are also used for the treatment of severe primary or reactivated herpes simplex virus infection. We distinguished between acyclovir treatment for herpes zoster and herpes simplex based on drug strength and package size in the prescription. However, because of the similarity of the valaciclovir and famciclovir treatment regimens for herpes zoster and herpes simplex, it was not possible to distinguish between them based on drug strength and package size for these drugs. Thus, cohort members were only categorised as exposed (from the date at which the prescription was filled) after filling their first prescription of acyclovir, with the specific strength of 800 mg in packages of 35 tablets. Cohort members with either a second prescription of acyclovir of this strength or package size, a first acyclovir prescription with a different strength or package size (consistent with treatment for herpes simplex), or a first prescription of valacyclovir or famciclovir (not possible to distinguish between zoster and simplex, as described above), were censored at the time at which the prescription was filled. We chose a conservative strategy for the definition of exposure in order to avoid exposure misclassification which may have had a minor impact on study power. Cohort members with no prior history of acyclovir, valacyclovir or famciclovir prescriptions were categorised as unexposed.

### Outcome – stroke

We used the National Patient Registry (NPR) to identify cases of stroke amongst cohort members. The NPR was established in 1977 and contains individual-level information on all hospitalizations including CRS-number, dates, duration and place of admission, and discharge diagnosis codes using the International Classification of Diseases (ICD), versions 8 and 10. In 1995, it was expanded to include information on outpatient hospital visits and emergency department visits. Using data from the NPR, we identified all cases of stroke and TIA (ICD-10 I60-64 and G45) between 1995 and 2008. The definition of stroke in this paper refers to the composite outcome of stroke and TIA. Persons registered in the NPR with an outcome diagnosis before the start of follow-up were excluded from the cohort using the ICD-8 codes 430–438 and the aforementioned ICD-10 codes.

To control for health care-seeking behaviour, we investigated another outcome which has not been associated with VZV, in an otherwise similar analysis. This outcome was defined as forearm, femur, and lower leg fractures using the ICD-8 codes 813, 820, 821, and 823, and the ICD-10 codes S52, S72 and S82.

### Potential Confounders

We obtained information on acute myocardial infarction (ICD-8 410, ICD-10 I21) and atrial flutter/fibrillation (ICD-8 42973–42794, ICD-10 I48) from the NPR, highest obtained educational level from Statistics Denmark, and information on cancer diagnoses from the Danish Cancer Registry. Information on antihypertensive drugs (ATC C03, C07, C09), drugs used in the treatment of dyslipidaemia and atrial flutter/fibrillation (ATC B01AA, B01AC, C01AA, C01BC, C01BD, C07AA, C08DA), and immunosuppressive drugs (ATC H02AB, A07EC, L04) was obtained from the NRMPS. We did not have information on risk factors such as smoking, untreated hypertension, obesity or family history of cerebrovascular disease.

### Statistical Analysis

Members of the cohort were followed from January 1, 1995, or their 18^th^ birthday, whichever came later, until the first of the following: filling of a second acyclovir prescription or an acyclovir prescription of another strength or package size than the recommended treatment for herpes zoster, filling of a valacyclovir or famciclovir prescription, HIV-diagnosis (because this disease increases the risk of herpes zoster), death, emigration, disappearance, outcome, or end of follow-up (December 31, 2008). Thus, persons were categorised as unexposed until they received the specific prescription for herpes zoster treatment after which they were considered exposed. A person that received the specific prescription for herpes zoster treatment followed by another antiviral prescription was only at risk as exposed until the second prescription, at which point the individual was censored. We used log-linear Poisson regression to calculate incidence rate ratios (IRRs), hereby comparing the rate in exposed cohort members with the rate in unexposed members. All IRRs were adjusted for age, sex and calendar period. In additional analyses, adjustments were made for highest obtained educational level (categories: primary school, secondary school, vocational or short tertiary education, medium tertiary education, long tertiary education), history of acute myocardial infarction diagnosis (yes, no), history of atrial flutter/fibrillation diagnosis (yes, no), treatment with selected cardiovascular drugs (yes, no), use of immunosuppressive drugs within the last 6 months (yes, no), and any cancer diagnosis within the last 6 months (yes, no). All variables except sex were treated as time-dependent variables. All tests were likelihood ratio homogeneity tests. All statistical analyses were conducted in SAS version 9.2.

## Results

The cohort consisted of 4,620,980 individuals; 2,278,782 men and 2,342,198 women. We identified 117,926 exposed individuals, of whom 4876 developed a stroke during the follow-up period. A total of 230,341 persons were diagnosed with stroke during 52,862,971 person-years of follow-up.

**Figure 1:**
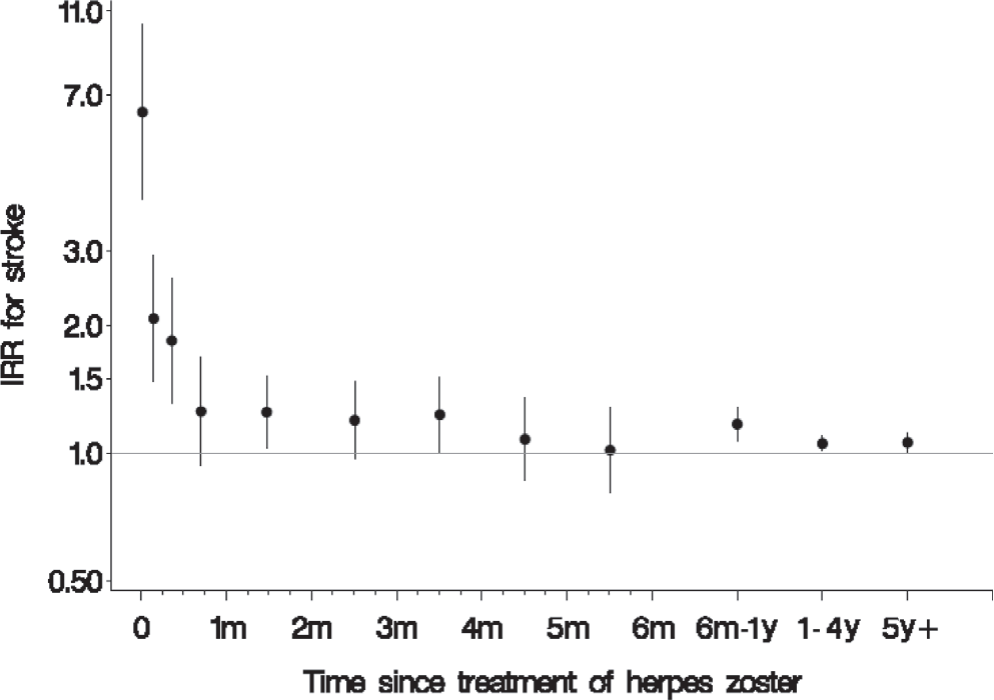
Incidence rate ratio (IRR) of stroke with 95% confidence intervals in individuals receiving antiviral treatment for herpes zoster by time since treatment compared with individuals with no antiviral treatment in Danish adults 1995–2008. The 12 dots represent point estimates for days 0 and 1–6; weeks 1 and 2–3; months (m) 1, 2, 3, 4, and 5; and the intervals 6 months to just below 1 year (y), 1 to 4 years, and 5 years or more.

Figure 1 shows the IRR of stroke amongst exposed compared with non-exposed individuals, by time since exposure in the exposed group. The IRR was particularly high within the first two weeks and remained slightly elevated throughout the first year, after which it decreased. The IRR of developing stroke within the first year of exposure was 1.21 (95% CI 1.14–1.29).

Table 1 presents the IRRs of stroke by history of antiviral treatment for herpes zoster by sex, age at the time of exposure, and time since exposure. Individuals that had received treatment for herpes zoster had a 2.27 (95% CI 1.83–2.82) fold higher risk of stroke during the first two weeks following treatment when compared to individuals that had no history of herpes zoster treatment. Between two weeks and one year after herpes zoster, the overall risk remained slightly elevated (IRR 1.17, 95% CI 1.09–1.24) but after one year, the overall risk did not appear to be much higher amongst exposed persons when compared with unexposed persons (IRR 1.05, 95% CI 1.02–1.09).

**Table 1:** Incidence rate ratio (IRR) of stroke in individuals receiving antiviral treatment for herpes zoster by time since treatment compared with individuals with no history of antiviral treatment in Danish adults 1995–2008.

Time since herpes zoster treatment												
	<14 days				14 days –1 year				>1 year			
	Years*	Cases	IRR (CI)¤	p-value#	Years*	Cases	IRR (CI)¤	p-value#	Years*	Cases	IRR (CI)¤	p-value#
Overall	5	83	2.27 (1.83–2.82)		100	931	1.17 (1.09–1.24)		413	3862	1.05 (1.02–1.09)	
Sex				0.71				0.72				0.49
Men	2	37	2.38 (1.72–3.29)		40	401	1.18 (1.07–1.30)		166	1613	1.04 (0.99–1.09)	
Women	3	46	2.19 (1.64–2.92)		60	530	1.15 (1.06–1.26)		247	2249	1.06 (1.02–1.11)	
Age at time of herpes zoster treatment				0.08				0.0002				0.002
<40 years	1	2	5.52 (1.38–22.1)		21	22	2.71 (1.79–4.12)		94	68	1.27 (1.00–1.61)	
40–59 years	2	14	3.77 (2.23–6.37)		30	118	1.40 (1.16–1.67)		137	651	1.18 (1.09–1.28)	
≥60 years	2	67	2.06 (1.62–2.62)		49	791	1.12 (1.05–1.20)		182	3143	1.03 (0.99–1.06)	

* Person years in thousands.

¤ Adjusted for age, sex, and calendar period.

# Test for homogeneity.

Men and women had a similar increased risk within the first two weeks of treatment, (IRR 2.38 vs. 2.19, p = 0.71), between two weeks and one year after treatment (IRR 1.18 vs. 1.15, p = 0.72) and after one year (IRR 1.04 vs. 1.06, p = 0.49). We found an overall increased risk of stroke regardless of age at treatment, but the risk decreased with increasing age, both within the first two weeks of exposure as well as after one year. However, the difference in risks between age groups within the first two weeks was not significant (p = 0.84) whereas the difference between two weeks and one year (p = 0.0002), and after one year were significant (p = 0.002). To further evaluate the multiplicative interaction with age we also estimated the mean rate in the three age groups: <40 years: 2.0 per 10,000 person-years, 40–59 years: 11.8 per 10,000 person-years, and ≥60 years: 30.2 per 10,000 person-years.

We adjusted the overall effect within the first two weeks for the following potential confounders but found no important changes in risk estimates: atrial flutter/fibrillation (IRR 2.23, 95% CI 1.80–2.76), acute myocardial infarction (IRR 2.25, 95% CI 1.81–2.79), treatment with drugs used for atrial flutter/fibrillation (IRR 2.19, CI 1.77–2.72), treatment with antihypertensives (IRR 2.20, 95% CI 1.77–2.73), treatment with drugs used for dyslipidaemia (IRR 2.26, 95% CI 1.82–2.80), cancer (RR 2.26, 95% CI 1.82–2.80), and treatment with immunosuppressive drugs (IRR 2.27, 95% CI 1.83–2.81). The estimates were also adjusted for highest obtained educational level, with no effect on the results. Similarly, adjusting for these potential confounders had no effect on the estimates for the time periods between two weeks and one year, and for more than one year.

To investigate the potential influence of health care-seeking behaviour, parallel analyses were conducted using forearm, femur, and lower leg fractures as outcomes. No markedly increased risk was seen among patients receiving treatment for herpes zoster within the first two weeks after treatment (IRR 1.03, 95% CI 0.79–1.35), between two weeks and one year (IRR 1.08, 1.02–1.14), and after one year (IRR 1.03, 95% CI 1.00–1.05) after having herpes zoster when compared to unexposed persons. These results were not modified by age, sex, or time since having herpes zoster. We investigated the IRRs for the first two weeks, between two weeks and one year, and more than one year after having herpes zoster in herpes zoster patients compared with others in three different age-groups. For individuals under the age of 40, the three IRRs were 0.29 (95% CI 0.04–2.05), 0.83 (95% CI 0.64–1.07) and 0.89 (95% CI 0.79–1.00), for individuals between the age of 40 and 59 they were 0.61 (95% CI 0.25–1.47), 1.12 (95% CI 0.97–1.28) and 1.11 (95% CI 1.04–1.18), and for individuals aged 60 or above they were 1.19 (95% CI 0.89–1.58), 1.10 (95% CI 1.03–1.17) and 1.02 (95% CI 0.98–1.05).

## Discussion

This nationwide cohort study found an increased risk of stroke after herpes zoster. This association was particularly pronounced during the first 14 days following herpes zoster and continued to be strong for more than three months. To our knowledge, this study is the largest study to date that examines the association between herpes zoster and stroke, and the first epidemiologic study to examine the time lag between herpes zoster episode and development of stroke in a large cohort.

Our findings are in line with two studies from Taiwan that reported a 31% increased risk of stroke the first year after herpes zoster and a 450% increased risk the first year after herpes zoster ophthalmicus. [5], [6] This study also confirms the short-term effect described in previous case reports and reviews. [7], [8], [11] It expands on previous reports by providing a detailed analysis with respect to time since herpes zoster infection, including an analysis of the long-term risk following infection. The risk was increased by 127% during the first two weeks, 17% between two weeks and one year after herpes zoster, and 5% after one year. The association was observed in both young and old age groups, with the highest increased risk observed in young patients.

The short-term effect may be explained by a productive viral infection that damages and weakens the walls of cerebral arteries, resulting in thrombosis, occlusions, infarctions, aneurysms or haemorrhage; significant changes have been identified on brain imaging and angiograms in patients with stroke occurring after herpes zoster.[3], [6], [9], [14]–[19] The observed association may also be the result of a transient prothrombotic condition caused by the viral infection. [8], [19].

Whilst the observed short term risk was high and may support a causal association, the long-term risk was less evident, with an overall increased risk of 5% after one year. The small increase is close to what was observed for the risk of selected fractures. We did however observe an increased long-term risk of stroke in patients younger than 60 years at the time of diagnosis of herpes zoster, which was not observed for selected fractures. Thus, a long-term effect, if any, is likely primarily to be expected in the younger age-groups. The long term-effect may be explained by a gradual atherosclerotic process induced by inflammation of the vessels caused by infection with herpes zoster. [5] Alternatively, the long-term effect in younger patients may simply be due to their lower age-specific incidence of stroke. We observed that the mean rate difference in younger patients was similar to that of older patients. This suggests that the additional increase in risk is the same regardless of age, but that this increase represents a higher relative increase in the younger age group.

We performed a similar analysis with fractures as an outcome, thereby investigating whether an increased risk of stroke was the result of a particular type of health care-seeking behavior amongst exposed persons. Had all persons receiving acyclovir had an increased tendency for subsequent hospital contact, we would have observed a similar increased risk of fractures. However, this was not the case, as neither the overall short-term risk nor the age-specific estimates for fractures after herpes zoster were as high as those for stroke after herpes zoster.

Our study was subject to several limitations. The use of acyclovir treatment as a proxy for herpes zoster may have resulted in false positive exposures as some people may have received it for herpes simplex. However, a misclassification of this nature would most likely lead to a bias towards the null. We minimized this potential bias by restricting the exposure to individuals that had only received one prescription of the recommended treatment regimen for herpes zoster (a 7-day course of 800mg tablets). By censoring persons who received a second acyclovir prescription, we may have censored persons with prolonged herpes zoster and a possibly greater risk of stroke. However, despite this, our results remained significant. Individuals who experienced VZV reactivation without the presence of the rash (zoster sine herpete), untreated herpes zoster, or a history of herpes zoster before the NRMPS was established would be defined as unexposed in our study; while this would bias estimates towards the null, individuals misclassified for these reasons consitute only a small fraction of the unexposed group. Therefore, this bias would be negligible. We investigated stroke risk in patients treated for herpes zoster; because antiviral treatment might reduce stroke risk conferred by the infection, our results may not apply to untreated herpes zoster. However, a previous study did not find a significant difference in stroke development between patients that had received antiviral treatment and those that had not. [6] We adjusted for a number of important potential confounders, but were unable to account for untreated cardiovascular risk factors, obesity, smoking and family history. In the event of differential prevalence of one or several of these factors in the exposed and unexposed groups, confounding could have been introduced; this could potentially bias results in either direction. However, adjustment for e.g. myocardial infarction, antihypertensive medication use, and use of lipid-lowering drugs had no impact on the main estimate. This argues against cardiovascular risk factors being strong confounders of the association between herpes zoster and stroke. Finally, the date of filling the prescription may not be the precise date of onset of herpes zoster, a misclassification that would underestimate the short-term effect. However, antiviral therapy is recommended within 3 days of disease onset, [20] and the date of filling the prescription is therefore likely close to the date of onset of herpes zoster.

Our study took advantage of the person-identifiable and mandatory registration of demographic and healthcare information in Denmark. It was based on a large cohort consisting of all adults in Denmark during a 14-year period, making selection bias unlikely, and we avoided loss to follow-up by using up-to-date vital status information from the Danish Civil Registration System. As the study was based on three different nationwide registers, it allowed for independent ascertainment of demographic information, exposure (prescription) information and stroke diagnosis, so a significant influence by differential misclassification of the exposure and outcome and by recall bias was avoided.

In conclusion, we found an increased risk of stroke following herpes zoster, that was most pronounced in the first 3 months following the exposure. A long term effect, if any, was only observed for persons who developed herpes zoster at a younger age.
